# Anatomical, Physiological, and Molecular Imaging for Pancreatic Cancer: Current Clinical Use and Future Implications

**DOI:** 10.1155/2015/269641

**Published:** 2015-06-04

**Authors:** John Chang, Donald Schomer, Tomislav Dragovich

**Affiliations:** ^1^Department of Radiology, Banner-MD Anderson Cancer Center, Gilbert, AZ 85234, USA; ^2^Department of Oncology, Banner-MD Anderson Cancer Center, Gilbert, AZ 85234, USA; ^3^Department of GI Medical Oncology, University of Texas MD Anderson Cancer Center, Houston, TX 77030, USA

## Abstract

Pancreatic adenocarcinoma is one of the deadliest human malignancies. Early detection is difficult and effective treatment is limited. Verifying the presence of micrometastatic dissemination and vessel invasion remains elusive, limiting radiological staging once this diagnosis is made. Diagnostic imaging provides independent tools to evaluate and characterize the biologic behavior of pancreatic cancer. Conventional anatomic imaging alone with either CT or MRI yields useful information on organ involvement but is limited in providing molecular and physiological information. Molecular imaging techniques such as PET or MRS provide information on metabolic and signaling pathways. Advanced MR sequences that target physiological parameters expand imaging options to characterize these tumors. By considering the parametric data from these three imaging approaches (anatomic, molecular, and physiological) we can better define specific tumor signatures. Such parametric characterization can provide insight into tumor metabolism, cellular density, protein expression, focal perfusion, and vascular permeability of these tumors. Radiogenomics research has already demonstrated ability to obtain information about cancer's genotype and phenotype; this is without invasive procedures or surgery. Further advances in these areas of experimental imaging hold promise to enable future clinical advances in detection and therapy of pancreatic cancer.

## 1. Introduction

*Overview Pancreatic Cancer*. Pancreatic adenocarcinoma is fourth leading cause of cancer deaths in the US, reflected by estimated 43,920 new cases and resulting in 37,390 deaths in 2013 [1]. Median survival time (SEER data) for pancreatic cancer is less than one year (for all stages), and mortality rate has remained unchanged in the past decade [2]. Very few patients are diagnosed early enough to be considered for a surgery and of them less than 20% are alive at 5 years. For patients with advanced and metastatic disease the median survival with therapy ranges between 6.1 and 11 months [3]. Recently, a combination of nab-paclitaxel and gemcitabine extended overall survival above that of gemcitabine alone, 8.5 months versus 6.7 months [4]. Another multidrug combination, FOLFIRINOX, has demonstrated considerable activity in a population of fit patients (median OS of 11.2 months) but the regimen was associated with some significant toxicities [5]. In the second-line therapy setting, effective options are even more limited, with very few durable responses [3, 6].

Advanced gene sequencing technology with high-throughput profiling platforms as well as molecular profiling provides potentially valuable prognostic and predictive tools as well as actionable targets. However, routine clinical translation is limited by the significant mutational heterogeneity in pancreatic cancer. Pancreatic cancer gene sequencing revealed the heterogeneity of molecular pathways disruption in pancreatic cancers [7]. A paper by Jones et al. shows that there is multiplicity of specific gene mutations across pancreatic adenocarcinomas. However, most genes are mutated only in a small subset of cancers but there is a multiplicity of affected signaling pathways. Thus, it is very unlikely that targeting of single gene alteration will be sufficient to derail cancer cell growth and/or ability to metastasize. While targeting the individual driver mutations may prove to be futile in pancreatic cancer, focus on targeting commonly deranged pathways such as metabolic pathways, neoangiogenesis, cell cycle regulation, and DNA repair pathways may be the more effective strategy [8].

Surgical resection in combination with adjuvant therapy remains the only way to achieve cure, but only minority of patients are long term survivors. This underscores the need for earlier diagnosis, better preoperative staging, and more effective systemic therapy. Standard surgical approaches include pancreaticoduodenectomy (Whipple's stomach- or pylorus-preserving) for tumors of the pancreatic head and distal pancreatectomy for tumors arising in the body or tail of the pancreas [11]. Pancreatic adenocarcinomas tend to have a distinct locally invasive growth pattern invading regional blood vessels, such as the portal vein or the superior mesenteric vein. Tumors encasing the superior mesenteric artery or the celiac vessels are considered unresectable. Only a small fraction of locally advanced tumors can be downstaged by chemoradiation therapy to become resectable (borderline resectable disease) as follows.

*Criteria for Borderline Resectable Pancreatic Adenocarcinoma [12]*

1. SMA abutment,
2. short segment abutment or encasement of celiac artery,
3. short segment abutment or encasement of common hepatic artery,
4. reconstructable occlusion of SMV-portal vein confluence.

It is very difficult to predict surgical resectability even with most advanced conventional imaging modalities. Some of the key challenges for clinicians diagnosing and treating pancreatic cancer are as follows: (1) difficulty in early detection, characterization, and localization of primary tumor; (2) the presence of severe desmoplastic stromal reaction that obscures tumor margins; and (3) inadequate ability to determine the response to therapy. In this paper, we provide an overview of the current status of clinical, physiological, and molecular imaging as applicable to pancreatic cancer and outline directions for future integration of these imaging modalities in clinical practice.

## 2. Current Clinical Anatomical Imaging with CT/MR

Imaging is critical to the care of pancreatic cancer patients at multiple stages of the disease. At initial diagnosis, imaging is used to stratify patients into resectable, borderline resectable, and nonresectable disease and to assess metastatic disease [18]. After treatment, patients are monitored for response to therapy, recurrent disease, or metastasis [18]. All of these tasks may be accomplished with anatomic imaging using either CT or MRI. Both imaging modalities exploit physical differences between various tissue types to create cross-sectional images that reflect a patient's specific anatomy. CT exploits differences in X-ray attenuation between tissue types to produce an anatomic representation. Conventional MRI creates anatomic images based on differential tissue relaxation times after RF excitation. Deviations from normal anatomy, including the presence of many tumors, are readily determined with either CT or MRI.

The primary modality for evaluating patients with pancreatic adenocarcinoma is CT. A dedicated pancreatic protocol CT scan maximizes the contrast differences between various tissues during the pancreatic and portovenous phases to improve the sensitivity for detecting vascular invasion, lymph node involvement, and liver metastasis [12, 18–21]. There is strong correlation between preoperative CT findings of vascular encasement and surgical findings [21]. Anatomic characterization with CT aids treatment planning in patients with borderline resectable disease since patients with short-segment vasculature encasement benefit from surgery if vascular reconstruction is feasible [12]. After the treatment, a CT scan is used to assess for therapeutic response and to detect metastatic disease or disease recurrence [20, 22].

MRI has shown similar sensitivity and specificity to CT for diagnosis and treatment planning in patients with pancreatic cancer [23–26]. In their meta-analysis, Bipat et al. found that CT is only slightly superior to MR in diagnosis of pancreatic cancer with a sensitivity of 91% (compare to 86% for MR) and specificity of 85% (compared to 82% for MR) [23]. CT and MRI are roughly equivalent for predicting resectability with sensitivity of 82 to 81% and specificity of 82 to 78% (CT to MR) [23]. Similar findings were reported by the Radiology Diagnostic Oncology Group in 1995 when the group compared MR and CT with regard to diagnosis, vascular invasion, and lymph node involvement [24]. MR may be more sensitive at detecting liver metastasis, but this does not appear to affect the overall accuracy of diagnosing metastatic disease [24]. MR does have one advantage over CT in that the study can be performed without intravenous contrast and still yield the high sensitivity, specificity, and accuracy, which may be useful in patients with renal insufficiency [24].

## 3. Current Clinical Molecular Imaging with FDG-PET

Depending on the radiotracer selection, positron emission tomography, integrated with computed tomography (PET/CT), provides molecular information that is mapped to an anatomic CT image. The most common tracer for oncology patients is 2-deoxy-2-[fluorine-18]fluoro-D-glucose (^18^F-FDG), since it identifies areas of high glucose metabolism. When the PET data is fused to CT images, those areas of high glucose metabolism can be identified and understood as discrete anatomic structures. Further CT provides a method to correct for positron attenuation through the body before it is measured at the detector. This correction is necessary to determine reproducible standard uptake values (SUV) of a specific anatomic feature. The SUV is the ratio of the radioactive concentration of a point or region of interest at some specific time to the injected activity at the same time point (original activity minus decay) divided by the patient's body weight. Therefore, PET/CT provides a quantitative measure of glucose metabolism mapped to anatomic features.

PET/CT is most useful for detecting metastatic dissemination of pancreatic cancer at initial diagnosis. Many reports examining the role of PET/CT scan suggest slightly higher specificity/accuracy for detection of pancreatic cancer (between 65 and 100%/69 and 91% compared to 65 and 100/64 and 85% for CT scan) [27–29]. However, this advantage disappears after adjusting for pretest probability [30]. The strongest rationale for using PET/CT in the initial evaluation of pancreatic adenocarcinoma is its ability to influence therapy choice. The PET/CT alters the initial therapy choice due to its high sensitivity for detecting metastatic disease (liver, peritoneal cavity), thus preventing unnecessary and morbid surgery [28, 29]. Review of the published studies showed that PET sensitivity and specificity for detecting liver metastasis can reach 85 and 97% and can alter management decisions in up to 27% of patients [28, 29]. In the surveillance setting, PET/CT more accurately identifies recurring lesions with detection rates reaching 96% while contrast enhanced CT detects less than 50% of recurrent lesions [28, 29]. Changes in FDG uptake before and after treatment can provide prognostic information [28, 31]. In the study by Topkan et al., cancers that demonstrate greater than 64% decrease in SUVmax following treatment had median survival times of 17 versus 11.2 months [31]. Cancers with higher baseline SUV tend to have greater metabolic response to neoadjuvant chemotherapy [32]. Also, patients with greater than 50% decrease in SUVmax following initial therapy have longer overall survival and progression-free survival [28, 31]. These results demonstrate that PET/CT can benefit pancreatic cancer patients by detecting metastasis and recurrences earlier and by providing prognostic/predictive information.

## 4. Translational Molecular and Physiological Imaging of Pancreatic Cancer

Modern MRI sequences can identify and localize data that might begin to define an individual tumor signature. Specific MRI pulse sequences can predict precapillary perfusion, vascular permeability, postcapillary drainage, and cellular density of a mass. MR spectroscopy can survey regions of interest within a mass for the presence and relative amount of certain molecular metabolites. Though it has largely found application in neuroradiology, blood oxygenation level dependent (BOLD) imaging may soon find application in the imaging of tumors anywhere in the body. Unlike CT imaging, which can only measure relative differences in electronic density, the above MRI techniques are largely uniquely different from one another. These differences result in mathematical independence of parameters that can be used to create unique tumor signatures.

By detecting the transfer and relaxation of hydrogen magnetic spins, MR is capable of detecting the various physiological changes in tissue. Cellular density is proportional to diffusion restriction so that a highly cellular tumor restricts water diffusion through the tumor mass [33–35]. MR perfusion measures the time profile of delivery of gadolinium contrast agent into and out of a region of interest. By using the appropriate arterial input function, one can estimate the proportion of vessels within the tumor mass as well as their permeability [36, 37]. These parameters may guide treatment determination and monitoring with antiangiogenesis agents. MR spectroscopy (MRS) evaluates the resonance frequencies resulting from electronic clouds around atoms. Differences in resonant frequencies result in measurably different signal allowing the identification of certain metabolites.  Table 1 provides an overview of certain MRS metabolites and their potential uses in body imaging. BOLD imaging exploits the differences in the paramagnetic properties oxyhemoglobin and deoxyhemoglobin, effectively using oxygenated hemoglobin as a contrast agent. This technique has long had application in neuroradiology and forms the basis of functional MRI (fMRI). Recently, BOLD technique has found application in body imaging [38, 39] and may be useful for differentiating areas oxygenated from relatively hypoxic areas within a tumor as hypoxic regions are known to resist standard treatment [40]. The parametric analysis of different MRI signals to characterize a signature of a tumor state is the goal of physiological imaging.

Where advanced MRI may provide physiological imaging parameters, PET/CT inherently measures molecular activity of specific pathways. The utility of FDG PET to characterize glucose metabolism is well understood and is widely used for cancer staging and surveillance. Non-FDG PET tracers have been tested in preclinical studies with animal models in order to visualize DNA synthesis, protein synthesis, protein expression, and tumor hypoxia. DNA and protein synthesis is evaluated through the use of ^18^F-FLT (fluorothymidine), ^11^C-Methionine, fluorotyrosine, and fluorotryptophan [41–47]. Protein expression and signaling pathways have also been targeted using either targeting peptides or antibodies conjugated to radiotracers, including epidermal growth factor receptor (EGFR), apoptosis activation (annexin V), and *σ* receptors [48–53]. Hypoxia is imaged with imidazole based agents (^18^F-MISO and ^18^F-FETNIM). This is very important because strong desmoplastic reaction around pancreatic cancer leads to decreased vascular density and induces hypoxia, which in turn increases cancer drug resistance [4, 54, 55]. Knowing these variable can help direct specific antihypoxia therapy to improve patient response to chemotherapy or radiation, such as dose-painting technique in radiation therapy [40].

**Figure 1:**
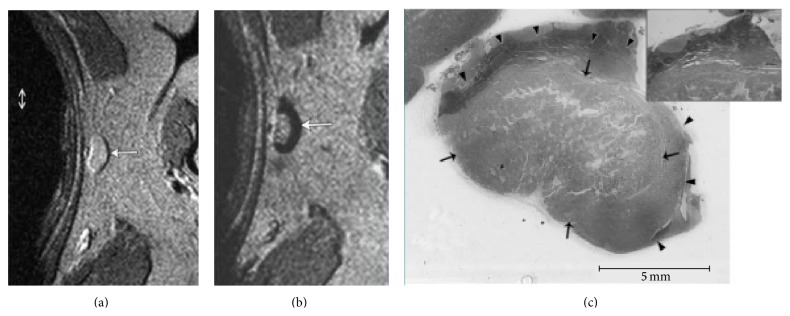
Iron oxide nanoparticles as lymphotropic agent. Normal portion of lymph node darkens after infusion of iron oxide nanoparticle while metastatic portion of the node does not darken (white arrow; (a) before and (b) after iron oxide nanoparticle administration). Corresponding sectioned lymph node showing metastasis marked by arrow and the normal portion marked by arrowheads (c). Reprinted with permission from [9].

Recent advances in MR imaging have enabled imaging of various molecular biomarkers* in vivo*. Much of this research is in its infancy and still far from clinical application for pancreatic cancer patient care [56]. Iron oxide nanoparticles are capable of tagging lymph nodes containing micrometastases; however, this technology is still awaiting regulatory approval (Figure 1) [56]. Other imaging agents have been developed to target specific cell membrane proteins, signaling, or metabolic pathways active in cancer cell. Examples include surface and matrix proteins such as prostate specific membrane antigen, integrin, and HER2 [56, 57]. Theoretically speaking, MR probes (such as gadolinium or iron oxide nanoparticles) can be linked to various ligands or antibodies in order to identify the proteins specific for cancer cell. However, obtaining regulatory approval for these molecular probes may be difficult and tedious process as seen in the case of the iron oxide nanoparticles.

Other functional imaging techniques based on MR are closer to clinical application. They include modalities such as hyperpolarized imaging, dynamic contrast enhancement imaging, and diffusion imaging. Hyperpolarized imaging requires specialized hyperpolarizer to enhance the magnetic spin of the molecules containing the detectable nucleus, which theoretically can limit the wide-spread clinical use. Carbon-13 containing metabolites such as pyruvate, bicarbonate, and glutamine can evaluate pathways that involve lactate, hydrogen ion, and glutamine metabolism [56]. Dynamic contrast enhancement and diffusion imaging employ commercially available MR sequences which make them more clinically relevant. Diffusion imaging has shown promise for improving pancreatic adenocarcinoma detection reaching detection rates greater than 95% [58–60].

Differentiating solid pancreatic mass from nonmalignant tissue can be improved by including lesion perfusion using either dynamic contrast enhancement or intravoxel incoherent motion techniques [59, 61]. Two known processes that can result in restriction of water diffusion include fibrosis as a result of desmoplastic reaction and proliferation of tumor cells, both of which occur within pancreatic tumor mass [59, 62–64]. The study by Wang et al. examined the ADC value of different grades of pancreatic cancer and fibrosis and found that poorly differentiated pancreatic cancer (with or without dense fibrosis) had similar ADC value as that of well and moderately differentiated pancreatic cancer with dense fibrosis (ADC~1.5 × 10^−3^ mm^2^/s) [64]. Tumor fibrosis may confound interpretation of diffusion findings as a low grade, fibrotic tumor may restrict diffusion to a greater degree than a higher grade, less fibrotic tumor. This is an inversion of the common concept where higher grade tumors typically restrict diffusion to a greater extent than lower grade tumors.

Multiphasic contrast enhanced MR studies can improve on the detection of the pancreatic cancer and provide potential differentiation between pancreatic adenocarcinoma and noncancerous tissues. In a small study using multiphasic contrast enhanced MR consisting of a cohort of 25 pancreatic cancers, arterial phase MR detected all 25, of which 14 could not be delineated on a contrast enhanced CT scans (Table 2) [13]. By using different DCE parameters (including rate transfer constant (Kep), peak time, maximum signal intensity, and extracellular-extravascular volume (Ve)), Liu et al. showed the potential of dynamic contrast enhancement (DCE) MR in differentiating pancreatic ductal adenocarcinoma (PDA) from non-PDA lesions [65]. Their study showed that Kep, peak time, and maximum signal intensity have the greatest ability to differentiate PDA from other masses in the pancreas and that these parameters correlated with tumor fibrosis and proliferation [66]. The greater fibrosis results in lower rate of transfer but greater extracellular volume fraction while greater cellular proliferation results in slower transfer and lower contrast enhancement [66]. In clinical trials, DCE MR has been used to assess tumor response to therapy by detecting early permeability changes 24 hours after initiation of therapy and following changes in permeability during the course of therapy [67]. Whether these changes translate into survival benefits remains to be tested in larger clinical trials.

## 5. Clinical Needs for Molecular Imaging and Imaging Biomarkers

### 5.1. Diagnosis: Differentiation of Pancreatic Adenocarcinoma from Mass Forming Pancreatitis

Mass forming pancreatitis (MFP) is a form of chronic pancreatitis and is difficult to differentiate from pancreatic adenocarcinoma [10]. The ability to differentiate these entities noninvasively could eliminate morbidities associated with biopsy and expedite appropriate therapy. Since MFP can demonstrate FDG avidity there are conflicting reports regarding the ability to differentiate MFP from pancreatic cancer with PET imaging. Review by Donswijk et al. established sensitivity and specificity of 94 and 90% in differentiating MFP from pancreatic adenocarcinoma for PET CT, compared to 82 and 75% for contrast enhanced CT [28]. Reports from Japanese groups indicate significant overlap in SUV values between MFP and pancreatic cancer [68, 69], suggesting that PET does not aid in differentiation between pancreatic adenocarcinoma and MFP. When used to differentiate masses, PET is most useful when uptake is at the extremes of metabolic activity. With CT and MR, studies have shown specific set of imaging features that favor MFP. These features and their odds ratio are shown in Table 3 [14, 70]. In addition, the multiexponential modeling of diffusion (intravoxel incoherent motion) images provides independent assessment of lesion perfusion without the need of IV contrast. Research using this technique shows that MFP tends to be better perfused than that of pancreatic cancer [61]. By using the combination of anatomical and physiological imaging features, it is possible to better determine the etiology of the pancreatic mass, although larger, multicenter study may be needed to determine the statistics of these imaging characteristics.

**Figure 2:**
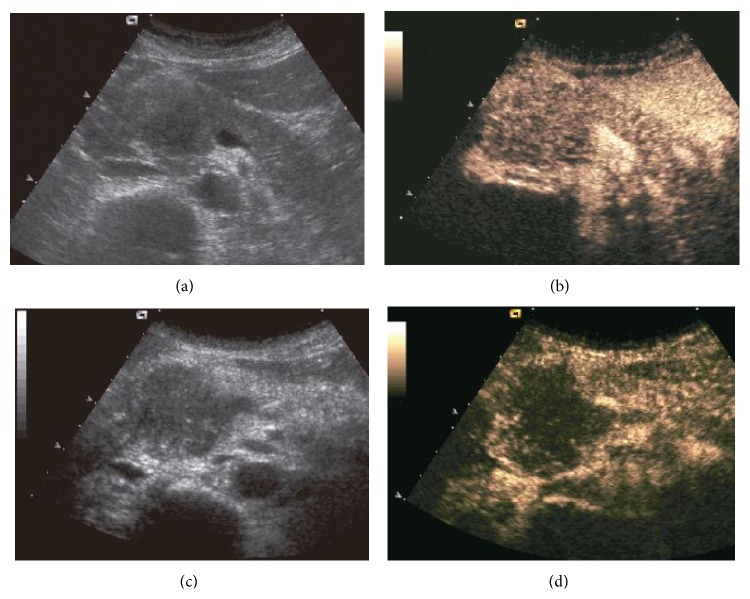
Changes in echogenicity following contrast administration. Microbubble contrast enhanced US study shows significant increase in echogenicity of MFP ((a) before contrast; (b) after contrast) while pancreatic adenocarcinoma shows minimal increase ((c) before contrast; (d) after contrast). Reprinted with permission from [10].

Another modality that can help with determining the nature and histology of the mass is microbubble-enhanced ultrasound but is still considered experimental due to the lack of an approved imaging agent in the United States at the current time. Following microbubble administration, MFP becomes either iso- or hyperechoic to the surrounding normal pancreatic parenchyma while pancreatic adenocarcinoma remains hypoechoic (Figure 2) [10]. This technique has a reported sensitivity and specificity of 88.6 and 97.8%. When microbubbles are bound to ligands that target either angiogenesis (VEGFR) or inflammation (cell adhesion molecules or integrins), they can distinguish tumor implants from inflammatory diseases [56]. However, additional research is needed to evaluate how specific each of these agents is in differentiating inflammatory and neoplastic pancreatic mass since angiogenesis and inflammation coexist in differing degrees in both MFP and pancreatic adenocarcinoma [71, 72].

### 5.2. Characterization: Imaging Markers of Molecular Mutations

Identifying imaging biomarkers of genotype and phenotype of cancer is the ultimate goal for oncological imagers as well as oncologists because this information can provide actionable targets without the need for tissue sampling. Research in the field of radiogenomics has shown potential of this approach to obtaining tumor genotype and phenotype. Much of this research was focused on better characterized cancer such as lung and brain where well-defined targets exist. In lung cancer, the presence of ground-glass opacity suggests >50% association with EGF mutation while growing solid component suggests >75% association with p53 expression (Table 4) [15]. The blood volume (BV) and extraction fraction (FE) of lung cancer obtained from dynamic contrast study correlate with clinicopathologic parameters, tumor hypoxia, and expression of glucose uptake transporter 1 (GLUT-1, Table 5) [16]. In glioblastoma multiforme, the imaging characteristics on T2-weighted and postcontrast images correlate with changes in various gene expressions [73]. Although this technique has yet to be applied to pancreatic cancer, it is conceivable that certain imaging characteristics of pancreatic cancer may suggest the genotype and phenotype of the cancer without direct biopsy.

In order to apply imaging biomarkers for identifying the genotype or phenotype of pancreatic cancer, clinically actionable mutations need to be identified. Although a large number of mutations exists in pancreatic cancer (including HER2/neu, K-RAS, Akt, p53, and p16INK4), clinical trials with erlotinib, cetuximab, and bevacizumab have yielded small clinical benefit [74, 75]. In preclinical trials with other epidermal growth factor (EGF) pathway inhibitors, a combinatorial approach of inhibiting the EGF family of tyrosine kinase inhibitors and gemcitabine have shown dramatic antitumor activity in pancreatic cancer xenografts [75]. At present, the exact clinical benefit of targeting these mutations remains to be established in human trials. As new clinical trials discover the importance of these pathways, imaging features could then be correlated with pathological findings to identify imaging features that suggest mutations favorable for treatment.

### 5.3. Treatment: Imaging Biomarkers for Predicting Response and Prognosis

The most important clinical question for an oncologist is how to predict whether or not the cancer will respond to the selected treatment and what (quality, duration) is the likely outcome of the treatment. Research has shown that pancreatic cancers that demonstrate strong postcontrast enhancement during the parenchymal phase (≥34 HU during pancreatic phase; ≥37 HU during portovenous phase; ≥47 HU during delayed phase) on a CT scan have much longer survival than those that do not (~13 versus 23 months of median survival) [76]. The lesions with greater delayed phase enhancement contain more fibrosis than those with less delayed phase enhancement and have lower probability of liver metastases than those with lower fibrosis (11.4% fibrotic content in patients with liver metastasis and 21.2% fibrotic content in patients without liver metastasis) [77]. This is due to the decreased vascular density, which is associated with greater tumor fibrosis [78]. Thus, patients with less fibrotic cancer may respond better to treatment but also have a greater likelihood of having metastatic disease at initial diagnosis. The current research has not fully evaluated other imaging biomarkers such as those for vascular encasement, vascular occlusion, celiac ganglion involvement, and tumor size, all potential predictors of response or prognostic factors.

### 5.4. Prevention: Screening

At present, effective therapies for pancreatic cancer are limited, short of complete resection in early stage disease. Hopefully, better understanding of the biology of pancreatic cancer will enable innovative treatment strategies that have far better outcome than FOLFIRINOX or gemcitabine plus nab-paclitaxel [4]. Until then, screening to identify premalignant lesions may provide best chance of cure. Patients that are at high risk for developing this disease, including patients with hereditary chronic pancreatitis, hereditary breast/ovarian cancer syndrome, and other hereditary neoplastic syndromes, are key populations to validate early pancreatic cancer screening methodology (Table 6) [17, 18, 26, 79]. Screening for pancreatic cancer in patients with hereditary pancreatitis has been recommended to start at age of 40 [18]. For patients with hereditary neoplastic syndromes or with first-degree relatives with pancreatic cancer, studies suggest that screening for these patients should begin at 40 years of age or 10 years prior to the age of onset for the affected relative as there is genetic anticipation [80]. However, insufficient data exists to suggest appropriate surveillance frequency [80]. The imaging modalities used for screening studies typically include CT, MR, or EUS (endoscopic ultrasound).

Recent report from the American Cancer of the Pancreas Screening Consortium on screening of 225 high-risk individuals discovered patients with cystic lesions (84), solid lesions (3), and dilated pancreatic duct (5) [81]. These cystic lesions were predominantly intraductal papillary mucinous neoplasms which have been shown to harbor high grade dysplasia even in lesions smaller than 3 cm in size [81]. The solid lesions were all found to be neuroendocrine tumors. These abnormalities increased in incidence with patient's age [81]. Of the modalities used for screening (CT, MR, and EUS), the rates for detecting the three types of lesions for CT, MR, and EUS were 11, 33.3, and 42.6%, making EUS the best modality for localized screening [81]. None of these techniques yield a satisfactory detection rate. However, MR avoids the invasive nature of EUS or the ionizing radiation of CT. Furthermore, MR can image the whole body with reasonable scan time due to recent advances in technology [82]. This ability to obtain full body screening for extrapancreatic tumors may be particularly valuable for patients with neoplastic syndromes. Although it is necessary to develop more sensitive diagnostic tests for pancreatic cancer it is important to balance the risks from high false-positive findings and benefits from identifying malignancy at an early stage [79].

## 6. Conclusion

In summary, pancreatic cancer is one of the most clinically challenging malignancies when it comes to prevention, detection, and therapy. Clinical challenges relate directly to genetic and pathophysiologic complexities of this disease as well as lack of validated preclinical models of pancreatic cancer for screening drug candidates. Strictly anatomic imaging technique such as CT and conventional MRI has utility in initial staging of patients. Molecular techniques such as PET and MRS add to our ability to evaluate the metabolic changes in individual tumors, providing information that improves prognostication and treatment planning. New MR techniques that provide information related to physiological parameters are very promising. There is hope that this advanced physiological imaging coupled with molecular and anatomical imaging may improve our ability to diagnose and follow patients with pancreatic cancer. Advancement of these imaging techniques will accelerate development of early diagnostic concepts and will greatly accelerate development of new and effective pharmacotherapy for this challenging disease.

## Figures and Tables

**Table 1:** Commonly evaluated MRS metabolites.

Metabolite	Resonance *v*	Significance
Lactate	1.3 ppm	Marker of anaerobic metabolism

Lipid	1.3 ppm	Marker of tissue damage with liberation of membrane lipids

Creatine	3.0 ppm	Energy storage: might serve as normalization baseline except in highly aggressive tumors where energy storage is used up

Choline	3.2 ppm	Marker for cell membrane turnover

**Table 2:** Enhancement pattern of pancreatic cancer on DCE MRI [13].

	Arterial Phase	Venous Phase	Equilibrium Phase
Hypointense	100%	80%	68%
Isointense	0%	20%	32%

**Table 3:** Odds ratio, sensitivity, and specificity of the significant MR findings in the diagnosis of mass-forming AIP [14].

MR findings	Sensitivity	Specificity	Odds ratio
Multiplicity	44.4	100	*∞*
Geographic shape	71.4	89.3	20.8
Delayed enhancement	71.4	78.5	9.2
Capsule-like rim enhancement	28.6	100	*∞*
ADC < 1.26 × 10^−3^ mm^2^/s	83.3	79.2	19.0
Skipped CBD stricture	33.3	100	*∞*
Skipped MPD stricture	44.4	100	*∞*

**Table 4:** Radiogenomics of lung cancer. Molecular mutations of lung cancer can be inferred from changes in the ground-glass and solid components of nodules. Reprinted with permission from [15].

Initial CT	Final CT		*N*	EGFR mutation	Positive p53
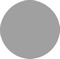	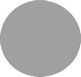	Persistent pure GGO	8	5 (63%)	0 (0%)

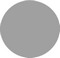	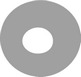	Change from pure to mixed GGO	3	1 (33%)	3 (100%)

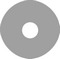	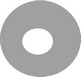	Mixed GGO with growth of solid component	4	2 (50%)	3 (75%)

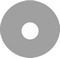	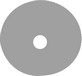	Mixed GGO with growth of GGO component	4	2 (50%)	0 (0%)

**Table 5:** Spearman rank correlations between DCE CT parameters and immunohistochemical markers of hypoxia in lung cancer. FE is inversely correlated with GLUT-1 expression while BV is inversely correlated with tumor hypoxia. Reprinted with permission from [16].

Parameter	Pimonidazole		Glut-1	
	*r* value	*P* value	*r* value	*P* value
FE	0.28	.12	−0.50	.002^*∗*^
BV	−0.48	.004^*∗*^	0.22	.19

Note. —Data are for specimens matched with DCE CT images (*n* = 35).

*∗* indicates a statistically significant difference.

**Table 6:** Relative risks for pancreatic cancer. List of relative risks of pancreatic cancer imparted from environmental and genetic risk factors. Reprinted with permission from [17].

Risk factor	Relative risk
Familial pancreatic cancer	
2 first-degree relatives affected	18
3 first-degree relatives affected	57
Hereditary pancreatic cancer syndromes	
BRCA2 mutation	5.9
Familial atypical multiple mole melanoma	16
Peutz-Jeghers syndrome	36
Hereditary pancreatitis	50
Cigarette smoking	
Positive family history of pancreatic cancer	3.7
Diabetes > 20 years	2

## References

[1] Siegel R., Naishadham D., Jemal A. (2013). Cancer statistics, 2013. *CA Cancer Journal for Clinicians*.

[2] National Cancer Institute Website (2008). *Joinpoint Regression Program*.

[3] Campen C. J., Dragovich T., Baker A. F. (2011). Management strategies in pancreatic cancer. *American Journal of Health-System Pharmacy*.

[4] von Hoff D. D., Ramanathan R. K., Borad M. J. (2011). Gemcitabine plus *nab*-paclitaxel is an active regimen in patients with advanced pancreatic cancer: a phase I/II trial. *Journal of Clinical Oncology*.

[5] Conroy T., Desseigne F., Ychou M. (2011). FOLFIRINOX versus gemcitabine for metastatic pancreatic cancer. *The New England Journal of Medicine*.

[6] Rahma O. E., Duffy A., Liewehr D. J., Steinberg S. M., Greten T. F. (2013). Second-line treatment in advanced pancreatic cancer: a comprehensive analysis of published clinical trials. *Annals of Oncology*.

[7] Jones S., Zhang X., Parsons D. W. (2008). Core signaling pathways in human pancreatic cancers revealed by global genomic analyses. *Science*.

[8] Dragovich T. (2012). Is there a case for personalized therapy of pancreatic cancer?. *Clinical Advances in Hematology & Oncology*.

[9] Memarsadeghi M., Riedl C. C., Kaneider A. (2006). Axillary lymph node metastases in patients with breast carcinomas: assessment with nonenhanced versus USPIO-enhanced MR imaging. *Radiology*.

[10] D'Onofrio M., Zamboni G., Tognolini A. (2006). Mass-forming pancreatitis: value of contrast-enhanced ultrasonography. *World Journal of Gastroenterology*.

[11] Warshaw A. L., Lillemoe K. D., Fernandez-del Castillo C. (2012). Pancreatic surgery for adenocarcinoma. *Current Opinion in Gastroenterology*.

[12] Katz M. H. G., Pisters P. W. T., Evans D. B. (2008). Borderline resectable pancreatic cancer: the importance of this emerging stage of disease. *Journal of the American College of Surgeons*.

[13] Chandarana H., Babb J., Macari M. (2007). Signal characteristic and enhancement patterns of pancreatic adenocarcinoma: evaluation with dynamic gadolinium enhanced MRI. *Clinical Radiology*.

[14] Hur B. Y., Lee J. M., Lee J. E. (2012). Magnetic resonance imaging findings of the mass-forming type of autoimmune pancreatitis: comparison with pancreatic adenocarcinoma. *Journal of Magnetic Resonance Imaging*.

[15] Aoki T., Hanamiya M., Uramoto H., Hisaoka M., Yamashita Y., Korogi Y. (2012). Adenocarcinomas with predominant ground-glass opacity: correlation of morphology and molecular biomarkers. *Radiology*.

[16] Mandeville H. C., Ng Q. S., Daley F. M. (2012). Operable non-small cell lung cancer: correlation of volumetric helical dynamic contrast-enhanced CT parameters with immunohistochemical markers of tumor hypoxia. *Radiology*.

[17] Klapman J., Malafa M. P. (2008). Early detection of pancreatic cancer: why, who, and how to screen. *Cancer Control*.

[18] Balachandran A., Bhosale P. R., Charnsangavej C., Tamm E. P. (2014). Imaging of pancreatic neoplasms. *Surgical Oncology Clinics of North America*.

[19] Brennan D. D. D., Zamboni G. A., Raptopoulos V. D., Kruskal J. B. (2007). Comprehensive preoperative assessment of pancreatic adenocarcinoma with 64-section volumetric CT. *Radiographics*.

[20] Lee E. S., Lee J. M. (2014). Imaging diagnosis of pancreatic cancer: a state-of-the-art review. *World Journal of Gastroenterology*.

[21] Lu D. S. K., Reber H. A., Krasny R. M., Kadell B. M., Sayre J. (1997). Local staging of pancreatic cancer: criteria for unresectability of major vessels as revealed by pancreatic-phase, thin-section helical CT. *American Journal of Roentgenology*.

[22] Bluemke D. A., Abrams R. A., Yeo C. J., Cameron J. L., Fishman E. K. (1997). Recurrent pancreatic adenocarcinoma: spiral CT evaluation following the Whipple procedure. *Radiographics*.

[23] Bipat S., Saffire S. K. S., Van Delden O. M. (2005). Ultrasonography, computed tomography and magnetic resonance imaging for diagnosis and determining resectability of pancreatic adenocarcinoma: a meta-analysis. *Journal of Computer Assisted Tomography*.

[24] Megibow A. J., Zhou X. H., Rotterdam H. (1995). Pancreatic adenocarcinoma: CT versus MR imaging in the evaluation of resectability—report of the radiology diagnostic oncology group. *Radiology*.

[25] Peddu P., Quaglia A., Kane P. A., Karani J. B. (2009). Role of imaging in the management of pancreatic mass. *Critical Reviews in Oncology/Hematology*.

[26] Sahani D. V., Shah Z. K., Catalano O. A., Boland G. W., Brugge W. R. (2008). Radiology of pancreatic adenocarcinoma: current status of imaging. *Journal of Gastroenterology and Hepatology*.

[27] de Gaetano A. M., Rufini V., Castaldi P. (2012). Clinical applications of 18F-FDG PET in the management of hepatobiliary and pancreatic tumors. *Abdominal Imaging*.

[28] Donswijk M. L., Hess S., Mulders T., Lam M. G. (2014). [18F]Fluorodeoxyglucose PET/computed tomography in gastrointestinal malignancies. *PET Clinics*.

[29] Higashi T., Saga T., Nakamoto Y. (2003). Diagnosis of pancreatic cancer using fluorine-18 fluorodeoxyglucose positron emission tomography (FDG PET)—usefulness and limitations in ‘clinical reality’. *Annals of Nuclear Medicine*.

[30] Orlando L. A., Kulasingam S. L., Matchar D. B. (2004). Meta-analysis: the detection of pancreatic malignancy with positron emission tomography. *Alimentary Pharmacology and Therapeutics*.

[31] Topkan E., Parlak C., Kotek A., Yapar A. F., Pehlivan B. (2011). Predictive value of metabolic 18FDG-PET response on outcomes in patients with locally advanced pancreatic carcinoma treated with definitive concurrent chemoradiotherapy. *BMC Gastroenterology*.

[32] Heinrich S., Schäfer M., Weber A. (2008). Neoadjuvant chemotherapy generates a significant tumor response in resectable pancreatic cancer without increasing morbidity: results of a prospective phase II trial. *Annals of Surgery*.

[33] Le Bihan D., Poupon C., Amadon A., Lethimonnier F. (2006). Artifacts and pitfalls in diffusion MRI. *Journal of Magnetic Resonance Imaging*.

[34] Padhani A. R., Koh D.-M. (2011). Diffusion MR imaging for monitoring of treatment response. *Magnetic Resonance Imaging Clinics of North America*.

[35] Taouli B., Koh D.-M. (2010). Diffusion-weighted MR imaging of the liver. *Radiology*.

[36] Brix G., Lucht R., Griebel J. (2006). Tracer kinetic analysis of signal time series from dynamic contrast-enhanced MR imaging. *Biomedizinische Technik*.

[37] Tofts P. S., Brix G., Buckley D. L. (1999). Estimating kinetic parameters from dynamic contrast-enhanced T1-weighted MRI of a diffusable tracer: standardized quantities and symbols. *Journal of Magnetic Resonance Imaging*.

[38] Chopra S., Foltz W. D., Milosevic M. F. (2009). Comparing oxygen-sensitive MRI (BOLD R2∗) with oxygen electrode measurements: a pilot study in men with prostate cancer. *International Journal of Radiation Biology*.

[39] Hernando C. G., Esteban L., Cañas T., van den Brule E., Pastrana M. (2010). The role of magnetic resonance imaging in oncology. *Clinical and Translational Oncology*.

[40] Dirix P., Vandecaveye V., De Keyzer F., Stroobants S., Hermans R., Nuyts S. (2009). Dose painting in radiotherapy for head and neck squamous cell carcinoma: value of repeated functional imaging with 18F-FDG PET, 18F-fluoromisonidazole PET, diffusion-weighted MRI, and dynamic contrast-enhanced MRI. *Journal of Nuclear Medicine*.

[41] Biswal S., Resnick D. L., Hoffman J. M., Gambhir S. S. (2007). Molecular imaging: integration of molecular imaging into the musculoskeletal imaging practice. *Radiology*.

[42] Dankerl A., Liebisch P., Glatting G. (2007). Multiple myeloma: molecular imaging with 11C-methionine PET/CT—initial experience. *Radiology*.

[43] Barwick T., Bencherif B., Mountz J. M., Avril N. (2009). Molecular PET and PET/CT imaging of tumour cell proliferation using F-18 fluoro-L-thymidine: a comprehensive evaluation. *Nuclear Medicine Communications*.

[44] Eriksson O., Selvaraju R., Borg B., Asplund V., Estrada S., Antoni G. (2013). 5-Fluoro-[*β*-^11^C]-L-tryptophan is a functional analogue of 5-hydroxy-[*β*-^11^C]-L-tryptophan *in vitro* but not *in vivo*. *Nuclear Medicine and Biology*.

[45] He S., Tang G., Hu K. (2013). Radiosynthesis and biological evaluation of 5-(3-[^18^F]fluoropropyloxy)-L-tryptophan for tumor PET imaging. *Nuclear Medicine and Biology*.

[46] Pimlott S. L., Sutherland A. (2011). Molecular tracers for the PET and SPECT imaging of disease. *Chemical Society Reviews*.

[47] Sahani D. V., Bonaffini P. A., Catalano O. A., Guimaraes A. R., Blake M. A. (2012). State-of-the-art PET/CT of the pancreas: current role and emerging indications. *Radiographics*.

[48] Hackel B. J., Kimura R. H., Gambhir S. S. (2012). Use of 64Cu-labeled fibronectin domain with EGFR-overexpressing tumor xenograft: molecular imaging. *Radiology*.

[49] Blankenberg F. G., Katsikis P. D., Tait J. F. (1999). Imaging of apoptosis (programmed cell death) with 99mTc annexin V. *Journal of Nuclear Medicine*.

[50] Vangestel C., Van De Wiele C., Mees G. (2012). Single-photon emission computed tomographic imaging of the early time course of therapy-induced cell death using technetium 99m tricarbonyl his-annexin A5 in a colorectal cancer xenograft model. *Molecular Imaging*.

[51] Chang A. J., de Silva R. A., Lapi S. E. (2013). Development and characterization of 89Zr-labeled panitumumab for immuno-positron emission tomographic imaging of the epidermal growth factor receptor. *Molecular Imaging*.

[52] Nayak T. K., Garmestani K., Milenic D. E., Baidoo K. E., Brechbiel M. W. (2011). HER1-targeted 86Y-panitumumab possesses superior targeting characteristics than 86Y-cetuximab for PET imaging of human malignant mesothelioma tumors xenografts. *PLoS ONE*.

[53] Niu G., Li Z., Xie J., Le Q. T., Chen X. (2009). PET of EGFR antibody distribution in head and neck squamous cell carcinoma models. *Journal of Nuclear Medicine*.

[54] Chang J. C., Gambhir S. S., Willmann J. K., Kratz F., Senter P., Steinhagen H. (2011). Imaging techniques in drug development and clinical practice. *Drug Delivery in Oncology: From Basic Research to Cancer Therapy*.

[55] Hu M., Xing L., Mu D. (2013). Hypoxia imaging with 18F-fluoroerythronitroimidazole integrated PET/CT and immunohistochemical studies in non-small cell lung cancer. *Clinical Nuclear Medicine*.

[56] Kircher M. F., Willmann J. K. (2012). Molecular body imaging: MR imaging, CT, and US. Part II. Applications. *Radiology*.

[57] Jang M., Yoon Y. I., Kwon Y. S. (2014). Trastuzumab-conjugated liposome-coated fluorescent magnetic nanoparticles to target breast cancer. *Korean Journal of Radiology*.

[58] Fukukura Y., Takumi K., Kamimura K. (2012). Pancreatic adenocarcinoma: variability of diffusion-weighted MR imaging findings. *Radiology*.

[59] Lemke A., Laun F. B., Klau M. (2009). Differentiation of pancreas carcinoma from healthy pancreatic tissue using multiple b-values: comparison of apparent diffusion coefficient and intravoxel incoherent motion derived parameters. *Investigative Radiology*.

[60] Park M. J., Kim Y. K., Choi S., Rhim H., Lee W. J., Choi D. (2014). Preoperative detection of small pancreatic carcinoma: value of adding diffusion-weighted imaging to conventional MR imaging for improving confidence level. *Radiology*.

[61] Kang K. M., Lee J. M., Yoon J. H., Kiefer B., Han J. K., Choi B. I. (2014). Intravoxel incoherent motion diffusion-weighted MR imaging for characterization of focal pancreatic lesions. *Radiology*.

[62] Klauss M., Gaida M. M., Lemke A. (2013). Fibrosis and pancreatic lesions: counterintuitive behavior of the diffusion imaging-derived structural diffusion coefficient d. *Investigative Radiology*.

[63] Muraoka N., Uematsu H., Kimura H. (2008). Apparent diffusion coefficient in pancreatic cancer: characterization and histopathological correlations. *Journal of Magnetic Resonance Imaging*.

[64] Wang Y., Chen Z. E., Nikolaidis P. (2011). Diffusion-weighted magnetic resonance imaging of pancreatic adenocarcinomas: association with histopathology and tumor grade. *Journal of Magnetic Resonance Imaging*.

[65] Liu K., Xie P., Peng W., Zhou Z. (2014). Assessment of dynamic contrast-enhanced magnetic resonance imaging in the differentiation of pancreatic ductal adenocarcinoma from other pancreatic solid lesions. *Journal of Computer Assisted Tomography*.

[66] Liu K., Xie P., Peng W., Zhou Z. (2015). Dynamic contrast-enhanced magnetic resonance imaging for pancreatic ductal adenocarcinoma at 3.0-T magnetic resonance: correlation with histopathology. *Journal of Computer Assisted Tomography*.

[67] Baker A. F., Adab K. N., Raghunand N. (2013). A phase IB trial of 24-hour intravenous PX-12, a thioredoxin-1 inhibitor, in patients with advanced gastrointestinal cancers. *Investigational New Drugs*.

[68] Kato K., Nihashi T., Ikeda M. (2013). Limited efficacy of ^18^F-FDG PET/CT for differentiation between metastasis-free pancreatic cancer and mass-forming pancreatitis. *Clinical Nuclear Medicine*.

[69] Matsumoto I., Shirakawa S., Shinzeki M. (2013). 18-fluorodeoxyglucose positron emission tomography does not aid in diagnosis of pancreatic ductal adenocarcinoma. *Clinical Gastroenterology and Hepatology*.

[70] Kim T., Murakami T., Takamura M. (2001). Pancreatic mass due to chronic pancreatitis: correlation of CT and MR imaging features with pathologic findings. *The American Journal of Roentgenology*.

[71] Hamilton T. D., Leugner D., Kopciuk K., Dixon E., Sutherland F. R., Bathe O. F. (2014). Identification of prognostic inflammatory factors in colorectal liver metastases. *BMC Cancer*.

[72] Smith G. R., Missailidis S. (2004). Cancer, inflammation and the AT1 and AT2 receptors. *Journal of Inflammation*.

[73] Jamshidi N., Diehn M., Bredel M., Kuo M. D. (2014). Illuminating radiogenomic characteristics of glioblastoma multiforme through integration of MR imaging, messenger RNA expression, and DNA copy number variation. *Radiology*.

[74] Cartwright T., Richards D. A., Boehm K. A. (2008). Cancer of the pancreas: are we making progress? A review of studies in the US Oncology Research Network. *Cancer Control*.

[75] Strimpakos A., Saif M. W., Syrigos K. N. (2008). Pancreatic cancer: from molecular pathogenesis to targeted therapy. *Cancer and Metastasis Reviews*.

[76] Fukukura Y., Takumi K., Higashi M. (2014). Contrast-enhanced CT and diffusion-weighted MR imaging: performance as a prognostic factor in patients with pancreatic ductal adenocarcinoma. *European Journal of Radiology*.

[77] Hata H., Mori H., Matsumoto S. (2010). Fibrous stroma and vascularity of pancreatic carcinoma: correlation with enhancement patterns on CT. *Abdominal Imaging*.

[78] Hattori Y., Gabata T., Matsui O. (2009). Enhancement patterns of pancreatic adenocarcinoma on conventional dynamic multi-detector row CT: correlation with angiogenesis and fibrosis. *World Journal of Gastroenterology*.

[79] Poruk K. E., Firpo M. A., Mulvihill S. J. (2014). Screening for pancreatic cancer. *Advances in Surgery*.

[80] Canto M. I. (2007). Strategies for screening for pancreatic adenocarcinoma in high-risk patients. *Seminars in Oncology*.

[81] Canto M. I., Hruban R. H., Fishman E. K. (2012). Frequent detection of pancreatic lesions in asymptomatic high-risk individuals. *Gastroenterology*.

[82] Attariwala R., Picker W. (2013). Whole body MRI: improved lesion detection and characterization with diffusion weighted techniques. *Journal of Magnetic Resonance Imaging*.

